# A lexical database of British Sign Language (BSL) and German Sign Language (DGS): Iconicity ratings, iconic strategies, and concreteness norms

**DOI:** 10.3758/s13428-025-02660-z

**Published:** 2025-04-09

**Authors:** Gerardo Ortega, Annika Schiefner, Nia Lazarus, Pamela Perniss

**Affiliations:** 1 Linguistics and Communication, University of Birmingham, Frankland Building Room G112, Edgbaston, Birmingham, B15 2 TT UK; 2 University of Cologne, Klosterstraße 79B, Brieffach: 14, 50931 Cologne, Germany

**Keywords:** Sign languages iconicity, Concreteness, Iconic strategies, Normed databases

## Abstract

Iconicity, understood as a resemblance relationship between meaning and form, is an important variable that has important psycholinguistic effects in lexical processing and language learning across modalities of language. With the growing interest in iconicity, clear operationalizations in terms of the different ways in which iconicity is construed and measured are critical for establishing its broader psycholinguistic profile. This study reports a normed database of iconicity ratings for the same concepts in British Sign Language (BSL) and German Sign Language (DGS). As a related dimension, we also report the type of iconic mapping strategy, i.e., a nominal variable that reflects the different ways in which signs make form-meaning associations for each sign. Finally, we include concreteness ratings for the same concepts. Data from deaf and hearing signers show that iconicity ratings are strongly correlated across both languages, with different distributions across the different strategies, and skewed towards the iconic end of the scale for all groups except German hearing non-signers. Concreteness ratings in BSL and DGS are correlated, though more weakly, and skewed towards the concrete end of the scale. Interestingly, this differs from findings for spoken languages, where concreteness ratings exhibit substantially stronger correlations and abstract concepts are more predominantly represented. We also find that iconicity and concreteness ratings have a moderate positive and strong positive correlation in BSL and DGS, respectively. These results will be useful in psycholinguistic research and highlight differences that can be attributed to the manual-visual modality of signs.

## Introduction

The discovery that sign languages have the same organisational structure as spoken languages opened unchartered territory in the mechanisms responsible for language processing and acquisition. Since then, an intriguing question that has occupied psycholinguists is whether linguistic forms expressed through the body are processed and acquired through the same underlying mechanisms as spoken languages. Research has found overwhelming evidence that regardless of modality, all language are governed by similar organisational principles (Sandler & Lillo-Martin, 2006) and are processed in similar ways as spoken languages (Emmorey, 2001). At the same time, research has shown that sign languages exhibit unique effects (e.g., visual memory, Keener & Gathercole, 2007; sign and word processing, Villameriel et al., 2019), which have been attributed to the visual modality (Perniss et al., 2015). Psycholinguistic studies of sign languages have thus been central to answering novel research questions, and testament to this is the growing number of studies exploring the link between sign languages and cognition (Emmorey, 2001), language evolution (Verhoef et al., 2015), the neurobiology of language (Xu et al., 2009), amongst others.

An interesting feature that has been observed in all sign languages and that has had an important impact in the language sciences is the high prevalence of iconicity, which can be understood as a resemblance relationship between meaning and form (Dingemanse et al., 2015; Ortega, 2017; Perniss et al., 2010). The notion of iconicity goes against the Saussurean view that the link between form (signifier) and meaning (signified) in language is arbitrary and thus lacking any evident connection (de Saussure, 1916). For decades, arbitrariness constituted the defining characteristic of language, whereas motivated or resemblance-based labels remained sidelined as a linguistic oddity that did not merit serious attention. However, the salience of sign iconicity has contributed to making it a central focus of enquiry. The field of sign linguistics has now a long-standing tradition on iconicity research from different theoretical frameworks and applying varied methodologies (Capirci et al., 2022; Cuxac & Sallandre, 2007; Demey et al., 2008; Taub, 2001).

Investigations of iconicity in the manual-visual modality have demonstrated its importance in language processing and learning. In sign languages, deaf signers are faster at detecting a sign when a previously presented picture highlights its iconic link, which has been interpreted as iconicity modulating lexical processing (Thompson et al., 2009; Vinson et al., 2015) (however, see Bosworth & Emmorey, 2010; Emmorey et al., 2020; Ortega & Ostarek, 2021). Iconicity has also been reported to facilitate sign production in hearing adults proficient in a sign language (i.e., bimodal bilinguals) (Pretato et al., 2018). Further, iconic signs seem to be the first to appear in the lexicon of deaf children (Caselli & Pyers, 2017; Thompson et al., 2012), which challenges earlier studies suggesting that infants lacked the world knowledge to make motivated form-meaning associations (Meier et al., 2008). Similarly, hearing adults at the earliest stages of sign learning also show a positive effect of iconicity in sign learning (Karadöller et al., 2024; Lieberth & Gamble, 1991; Mott et al., 2020). In the realm of gesture, studies have unravelled the positive effect of iconic gestures in the acquisition of a sign language as a second language (Ortega et al., 2019), as well as in the facilitation of communication in people with aphasia (van Nispen et al., 2016) and people with autism (Cairney et al., 2023). This overwhelming body of work tells us that when iconicity is incorporated into psycholinguistic enquiry, it reveals important effects not documented before. Unfortunately, although iconicity has been recognised as an important variable, the ability to document its psycholinguistic profile remains hampered by the lack of normed databases.

With the growing awareness that language is multimodal in nature (Vigliocco et al., 2014) and that iconicity is an important factor that modulates processing and learning (Perniss & Vigliocco, 2014), the attempts to define iconicity, operationalise it, and understand if and how people make form-meaning associations with iconic forms (Dingemanse et al., 2020) have become more widespread, across both spoken and signed languages. Some scholars have used their personal intuitions to determine the features that link a meaningful unit (speech, gesture, or sign) with a referent (Lepic & Padden, 2017). Forced-choice associations, whereby participants are asked to match an acoustic or visual prompt with a picture or word to establish form-meaning associations, continue to be prevalent, for example, matching the nonce words *maluma* or *takete* with round or pointy shapes (Ramachandran & Hubbard, 2001), or matching iconic vocalizations with one of many possible meanings (e.g., selecting the word ‘water’ for a recording of a gulping sound) (Perlman & Lupyan, 2018). The last few years have also witnessed a growing amount of attention to describing appropriate methodologies to establish the kinds of resemblance-based form-meaning associations that can be detected in speech, gesture, and sign (Dingemanse et al., 2020; Motamedi et al., 2020).

One of the most common techniques used to operationalise the investigation of iconicity in linguistics and psychology is iconicity ratings. In this task, participants are presented with linguistic stimuli, one at a time, and are asked to give a numerical value that reflects how well the stimulus represents its meaning. For example, participants could be presented with the English word ‘screech’ after which they select a number from 1 to 7 that best fits the word’s form-meaning relationship. The opposite ends of the scale represent low vs high iconic relationships (e.g., 1 arbitrary vs 7 iconic) and the numbers in between a gradation of this continuum. In our example, the word ‘screech’ evokes a shrieking sound so participants may be inclined to assign a high value. The word ‘menu’, in contrast, does not include any evident cue that could evoke its meaning so participants may give it a lower rating (Winter et al., 2023). Researchers have also made modifications to iconicity scales whereby numerical values do not go along a positive scale (1–7) but rather include positive (+) and negative numbers (–). The rationale is that positive values denote iconic relationships (i.e., the form reflects its meaning), negative values suggest that the form represents the opposite meaning, and zero reflects a neutral form-meaning association (Perry et al., 2017). The underlying assumption of all Likert scales is that graded properties can be assessed by means of values along a continuum, and as such, ratings are widely accepted as a measure to quantify the degree of iconicity.

While the investigation of sign languages (and gesture) has been critical to understanding the importance and prevalence of iconicity in language, norms for iconicity (as well as for other psycholinguistic variables) in the manual-visual modality are still rare. However, after nearly 70 years of documentation and description of the sign languages of deaf communities, the last ten years have witnessed a growing number of normed databases that are essential to carrying out controlled experiments of sign language processing and acquisition. These databases exist, for example, for American Sign Language (ASL) (Caselli et al., 2016; Mayberry et al., 2014), Spanish Sign Language (*Lengua de signos española, LSE*) (Gutierrez-sigut et al., 2015), British Sign Language (BSL) (Vinson et al., 2008), and German Sign Language (DGS) (Trettenbrein et al., 2021). These databases provide data regarding iconicity, frequency, and age of acquisition of signs, which are known to play a key role in language processes. They parallel databases that exist for traditional psycholinguistic research on spoken languages (Brysbaert et al., 2014a, b; Kuperman et al., 2012). However, they differ quite substantially in the number of signs for which ratings exist, ranging from the low hundreds to the low thousands. Even for the larger databases, the amount of available data lies far below that which is available for many spoken languages. In addition, and due to the absence of a conventional written form, sign databases sometimes provide detailed descriptions of the formational features of signs (i.e., location, movement, hand configuration, orientation, and non-manual components). Lexical databases of sign languages, thus, typically provide conventional measures for psycholinguistic research as well as features that are exclusive to the manual-visual modality.

The use of ratings and the treatment of iconicity as a gradient feature of signs is not the only way to operationalize iconicity. Another psychologically valid operationalization of iconicity is as a categorical property that can be classified into types. Different scholars have noted, for instance, that there are semiotic relations that fit within clearly defined boundaries such as imagistic vs diagrammatic or primary vs secondary (Dingemanse et al., 2020; Sonesson, 1994). Within the manual-visual modality, more specifically, researchers have observed that signs and gestures can create depictive links with a referent through different strategies or modes of representation (Hwang et al., 2016; Mandel, 1977; Müller, 2015; Padden et al., 2013). Based on these observations, researchers have developed categories to describe the ways in which signs and gestures can depict the visual features of a concept.

The *acting* strategy (also referred to as *handling*) represents bodily actions or actions associated with a referent. E.g., the concept ‘swimming’ can be represented by re-enacting the arms in motion as if swimming in a pool. The *entity* strategy does not represent the body but rather uses different hand and body configurations to represent the shape and motion of an object or living entity. E.g., a clawed open hand with wiggling fingers can represent ‘spider’. *Tracing* (or *drawing*) uses the fingers or palms of the hand to trace the outline of an object. E.g., an extended index finger tracing a horizontal circular shape can represent ‘lake’ or ‘pizza’. In *personification*, a (non-human) living or non-living entity is mapped onto the person’s body to recreate physical resemblance. E.g., extended flapping arms can be used to represent ‘bird’ or ‘butterfly’. *Representing* typically incorporates visual features of a tool as well as the bodily action used to manipulate it. E.g., ‘scissors’ can be represented with extended index and middle fingers with an open-close forward motion. These different representational strategies have been shown to characterise modes of expression and meaning-making processes across both sign and gesture studies, though different nomenclatures exist for the different strategies (Hou, 2018; Hwang et al., 2016; Müller, 2013; Padden et al., 2013).

The relevance of these different types of iconic representations has been attested in several domains. Studies have demonstrated that these types of representation are employed differentially and with important implications in linguistics, language learning, and evolution. For instance, Carol Padden and colleagues developed the notion of *patterned iconicity* whereby the use of particular iconic strategies is associated with particular groups of referents, e.g., hand-held objects (Padden et al., 2013), and which may be exploited to mark grammatical distinctions, e.g., a noun–verb distinction (Padden et al., 2015). The *acting* and *representing* strategies (*handling* and *entity* in their terminology) are systematically related to the expression of verbal and nominal concepts, respectively, in ASL, e.g., an extended index finger moving rapidly up and down in front of the mouth represents the noun TOOTHRBRUSH (*representing*), while a closed fist with the same motion represents the verb BRUSH-ONE’S-TEETH (*acting* strategy). There is also evidence that gesturers have a generalised use of iconic strategies, while deaf signers of American and Italian Sign Languages use specific strategies to refer to agentive and non-agentive events (Brentari et al., 2015). Some have argued that the use of these different iconic strategies is an important feature that can be used as a principle of typological categorization of the sign languages of the world (Nyst, 2016). Importantly, the approach of investigating types of iconicity has shown that not all strategies have the same weight but rather some are more favoured than others.

For instance, the *acting* strategy appears to be a particularly salient form of representation in the manual-visual modality. Research exploring the communicative potential of iconic gestures has found that gestures employing the *acting* strategy are more easily produced and comprehended by patients with aphasia (Van Nispen et al., 2017). When prompted to produce elicited silent gestures, Dutch speakers have been observed to have a strong preference for using the *acting* strategy, in particular when referring to actions and manipulable objects (Ortega & Özyürek, 2019a). Deaf children acquiring a sign language from birth have been reported to have a strong preference for action-based signs, and interestingly, their deaf caregivers align with this preference even when they use other non-action-based iconic forms for the same referents when interacting with other deaf adults (Ortega et al., 2017). This body of work suggests that looking at different types of iconic representation is important for a better understanding of how iconicity modulates cognitive operations and linguistic processes. Unfortunately, to date, an operationalization according to type of iconicity, in conjunction with operationalizations using ratings, is largely missing from the existing databases of iconicity norms.

In addition, while research on iconicity is growing, its relationship with other psycholinguistic variables, such as concreteness, is not well understood. Concreteness is defined as the degree to which a concept denoted by a word is perceptible to the senses (Brysbaert et al., 2014a, b). For example, ‘table’ would be an example of a concrete concept because it is a highly tactile entity that can be experienced through the senses. A concept like ‘religion’, in contrast, can be classified as abstract because it cannot be touched, smelled, heard, seen, or tasted and as such is detached from the sensory world. Concreteness is an important construct that is responsible for many psycholinguistic effects. For instance, words expressing concrete concepts are typically the first to be acquired by children (Clark, 2009) and are processed differently from words expressing abstract concepts (Kousta et al., 2011). These facilitatory effects have been explained in terms of words for concrete concepts having strong semantic links with networks of visual processing (Paivio, 1986).

Some have argued that sensory experience is also an important factor for a word’s degree of iconicity. It has been observed that words for concrete concepts tend to be perceived as more iconic arguably because iconicity is better suited to the depiction of sensori-motor experiences. Lupyan and Winter (2018) have found that abstract words tend to be rated as less iconic, and that this is one reason why we do not observe more iconic forms in language. Namely, language consists of mostly abstract concepts and iconicity is restricted to tangible, observable contexts. In their words, ‘abstractness resists iconicity’ and ‘… words prefer to be arbitrary’ because iconicity grounds concepts to specific sensory depictions (Lupyan & Winter, 2018; p. 7). These claims would suggest that sensory experience modulates the degree of concreteness and iconicity, and that these measures could potentially be highly correlated (i.e., concrete concepts are more likely to be more iconic).

These arguments have been made for spoken/written languages, like English, but do not necessarily apply to sign languages in the same way. As stated earlier, there is a high prevalence of iconicity in all documented sign languages, and importantly, both concrete *and* abstract concepts can be represented through iconic mappings (Taub, 2001). For instance, the BSL sign COURT traces the shape of a wig because barristers in the UK wear a wig in court. In this example, iconicity is used to represent a physical object (i.e., a wig), which is in turn (metonymically) associated with an abstract concept (i.e., court). Similarly, the abstract concept of ‘freedom’ in BSL is expressed by representing the release of handcuffed wrists. This embodied representation is experienced through the senses, and through (secondary) metaphorical/metonymic extension, it depicts a more abstract notion. Sign languages thus present an excellent test case to explore the relationship between iconicity and concreteness and identify whether the visual modality influences the perception of concreteness and how concreteness interacts with different iconic strategies.

The present paper reports ratings (iconicity, concreteness) and iconic strategies for two unrelated sign languages: British Sign Language (BSL) and German Sign Language (Deutsche Gebärdensprache – DGS). These norms stem from a larger project investigating the similarities and differences in the use of iconicity in these sign languages and the gestures produced by the local hearing communities. Comparative cross-linguistic studies are key to understanding how iconicity manifests in the different sign languages of the world and to gain insights into how it shapes the lexicon. The potential for iconicity in the visual modality is responsible for lexical similarity across sign languages, independent of any geographical or other historical relationships (Johnston & Schembri, 2007; McKee & Kennedy, 2000; Woll, 1984). For example, many sign languages represent ‘tree’ using an extended vertical arm with open hand to depict the trunk and branches of a tree (Klima & Bellugi, 1979). Lexical comparisons interested in establishing historical relatedness have avoided taking iconic signs into account for this reason (Ebling et al., 2015). Yet, the impact of iconicity on lexical similarity is so striking that it has been the specific focus of attention in other studies (Pietrandrea, 2002; Pizzuto & Volterra, 2000; Schiefner et al., submitted). The present comparison will allow us to better understand the role of iconicity in the lexicon by investigating the relationship between different psycholinguistic variables for the same concepts across BSL and DGS.

## The present study

This study aims to expand our current knowledge on the degree of iconicity, iconic strategy, concreteness, and how these variables interact with each other in two unrelated sign languages: BSL and DGS. This investigation consists of two parts. First, we carried out a sign elicitation task where deaf signers in the UK and Germany were asked to produce as many signs as possible related to a semantic category presented in their respective sign languages. Many studies have used translations of written words or adaptations of the Swadesh list to elicit and document the form of the core lexicon of various languages (Padden, 2015). We opted for the creation of a set of concepts that represented good exemplars across different semantic categories (e.g., food, sports) by members of the specific language communities under investigation. By using a free elicitation task starting from broad semantic categories in BSL and DGS, our database consists of signs produced by deaf signers that are likely to reflect signs that are most frequently used, culturally relevant, and that are not (rough) translations of a spoken/written word.

The resulting dataset of elicited signs was glossed and matched across BSL and DGS. We then selected concepts named in both languages that represented a spread across different semantic categories. This resulted in a total stimulus set of 234 signs matched across the two languages, which were then coded for iconic strategy (i.e., *acting, representing, entity, tracing, personification, deictic*). In order to obtain iconicity ratings, we re-recorded the final list of 234 signs and collected iconicity ratings on an online platform from deaf and hearing signers. In addition, we collected concreteness ratings from deaf signers and hearing non-signers (BSL and DGS) as well as hearing signers (only DGS) to gain insight into its relationship with iconicity and iconic strategies. The data available from this database consists of videos of BSL and DGS signs for the same concepts along with psycholinguistic measures (iconicity ratings, type of iconicity (i.e., iconic strategy), and concreteness).

## Methodology

As stated earlier, the study consists of two parts. First, (i) a sign elicitation task where deaf signers produced signs for various concrete and abstract semantic domains. The signs were then glossed and categorised according to their iconic strategy (or mode of representation). The second stage consisted of (ii) ratings tasks where deaf and hearing non-signers were asked to rate individual signs on the dimensions of iconicity and concreteness.

### Sign elicitation task

#### Participants

Deaf signers of BSL in the UK (*N* = 4, mean age = 42, range 37–47; gender: two women, two men) and deaf signers of DGS in Germany (*N* = 5, mean age = 38.2, range 25–54; gender: one woman, four men) took part in the task.^1^ Participants used their respective sign language during the task and received monetary compensation for their involvement in the study.

#### Procedure

Participants were invited to the lab and were seated in front of an experimenter, with a laptop on one side and a video camera in front of them. After reading and signing consent forms, participants were given an explanation of the task in BSL/DGS and were encouraged to ask any clarification questions. Once participants were comfortable to start the task, they pressed a key to see a set of signed instructions on the laptop. Participants were told they would see a series of signs naming various concrete and abstract semantic domains (e.g., TIME, OUTDOOR ACTIVITIES, TOOLS, ARTS, POLITICS) and their task was to generate as many signs as possible related to that semantic category. The video of the semantic category played on a loop while participants generated their signs. A total of 24 semantic categories were displayed. The researcher told them that there was no right/wrong answer and that we wanted their most intuitive responses. The interlocutor would acknowledge each sign rendition, but would not interfere when participants asked, for example, if a sign corresponded to a category or not. When participants could not generate any more signs, they could press the space bar on the keyboard to move on to the next semantic category.

#### Data analysis

All signed renditions were annotated using the linguistic annotator ELAN (Sloetjes & Wittenburg, 2018). Participants produced a total of 2940 tokens in BSL and 6902 tokens in DGS. We compared both datasets from British and German participants and matched concepts that had been produced in both languages and by all participants. This resulted in a total of 234 concepts that were matched across BSL and DGS. The goal was a total of ten concepts for the 24 semantic domains, however, not all domains yielded ten concepts matched across the two languages. The resulting concepts were classified according to their iconic strategies (i.e., type of iconic mapping or mode of representation) using the criteria below. The coding scheme included taxonomies from sign and gesture studies (Müller, 2013; Padden et al., 2015; Van Nispen et al., 2017), and was adapted from our past research (Ortega & Ozyurek, 2019; Ortega & Özyürek, 2019b). The full coding scheme with our operationalisation can be found in the OSF repository.^2^

1. *Acting*.^3^ The body represents the body, and thus the sign represents bodily actions, how an object is manipulated, or actions associated with the referent. The DGS sign BREASTROKE represents someone swimming in this style.
2. *Representing*. The hand configuration represents the visual features of an object while incorporating features of how the object is manipulated (e.g., a V-handshape representing the sign SCISSORS).
3. *Entity.* The hands represent an object as a whole, in particular, objects not manipulated with the hands (e.g., the BSL sign PLANE is executed with a closed fist with extended thumb and pinky finger).
4. *Personification*. A non-human body (animal or object) is mapped onto the body of the signer such that their body parts align with the referent (e.g., the BSL sign MONKEY re-creates a monkey scratching its chest).
5. *Tracing*. The hands or fingers describe in space the outline of a referent (e.g., the DGS sign HOUSE traces in space the pointy roof of a house).
6. *Deictic*. This category includes signs that are not iconic strictly speaking but exhibit an indexical relationship with the referent (e.g., the BSL sign NOSE consists of an index finger pointing to the signer’s nose).
7. *Other*. Here we included signs that did not fit in any of the previous categories, and for which an iconic motivation was not apparent or was too obscure to be classified with certainty.

Participants’ sign renditions for the selected concepts were categorised for iconic strategy in a multi-stage process. Signs were first classified by a researcher fluent in the respective sign language. Once all signs were categorised aligning with the coding criteria, the whole team discussed inconsistencies and problematic examples. Finally, two team members checked one last time all signs and their categorisation until signs across both languages until there was full agreement that all signs were consistently coded.

All signs were then re-recorded professionally to develop stimulus materials for the ratings tasks. Data collection was carried out using the online platform LimeSurvey (2012). Participants for all rating tasks were recruited through video flyers, posters, and social media. Those participants who expressed interest in our study and registered to take part received a web link that would take them to one of the online tasks (iconicity or concreteness ratings). The two tasks described below started with a background questionnaire where participants provided information about their demographic and linguistic backgrounds.

### Iconicity ratings tasks

#### Participants

In the UK, a total of 32 raters completed^4^ the iconicity rating task (mean age 23.09, range 18–36; 26 women, six men). Of these, 17 were hearing non-signers and 15 were deaf signers. In Germany, a total of 53 raters completed the task (mean age 31.49, range 19–60; 39 women, 14 men). Twenty-three of these were non-signers and 30 were signers, of which 23 were deaf and seven were hearing.

#### Procedure

After completing background questionnaires and receiving instructions in BSL/DGS, participants started the task. They were informed that they would be presented with individual signs under which there was a slider with values ranging from 100 (arbitrary) to 700 (iconic). At the beginning of each trial, the cursor was located in the middle of the slider and participants were instructed to select a number along the continuum, which reflected how well the sign represented its meaning. English and German translations of the instructions can be found in the Appendix. Videos of the signed instructions can be found in our OSF repository^2^.

Participants were given three examples as part of the instructions to get familiarised with the task (these were not included in the actual experiment). In addition, the first five items were identical for all participants, allowing for further quality controls. After completion of the practice trials, they advanced to the task. Given the length of the task, and in order to avoid participant fatigue, participants were allocated to one of five lists with 46–47 signs each (i.e., each participant rated only a subset of all 234 signs). Signers could participate for a second or more blocks if they chose to.

As signers were presented with the signs without target translations, we included a sign verification task after the ratings. Participants once again saw all the signs in their list, one at the time, and were instructed to type in the English/German translation of the sign. It is well documented that there is a large lexical variation in all sign languages (Stamp et al., 2014), so it may have been possible that our tasks included signs that were different to participants’ known variant. This additional check allowed us to verify that participants were indeed rating the intended sign and its corresponding meaning. Items for which translations did not match the intended meaning were excluded from further analysis. This resulted in a median number of 13 ratings for BSL signs (range 4–32, Q1 = 9, Q3 = 19) and 19 ratings for DGS signs (range 7–52, Q1 = 16, Q3 = 22).^5^

### Concreteness ratings

#### Participants

In the UK, 15 deaf signers contributed to the concreteness rating task (mean age 35.07, range 20–57; ten women, five men). In Germany, 18 deaf signers contributed to the concreteness rating task (mean age 35.28, range 20–51; 16 women, two men; 18 deaf).

#### Procedure

The experimental setup and procedure for the concreteness ratings tasks mirrors closely the iconicity ratings task. After signing consent forms and completing background questionnaires, deaf BSL/DGS signers were allocated to one of five lists with 46–47 signs each. They were told that they should use the slider under each sign to select a value between 100 (concrete) and 700 (abstract) that best reflected the sign’s degree of abstractness/concreteness. We followed closely research on written/spoken languages and adapted our instructions to mirror those of previous studies researching concreteness (Brysbaert et al., 2014a, b). English and German translations of the instructions can be found in the Appendix. These can also be found in our OSF repository (see link in footnote 2) along with videos of the signed instructions. Participants in this task also completed a sign verification task where they saw the same set of signs and had to type in the meaning. Ratings of the responses that did not match the intended meaning were discarded and not included for further analysis. This resulted in a median number of six ratings for BSL signs (range 1–14, Q1 = 3, Q3 = 7) and seven ratings for DGS signs (range 2–18, Q1 = 6, Q3 = 8).

## Results

### Iconicity ratings for BSL and DGS

**Fig. 1:**
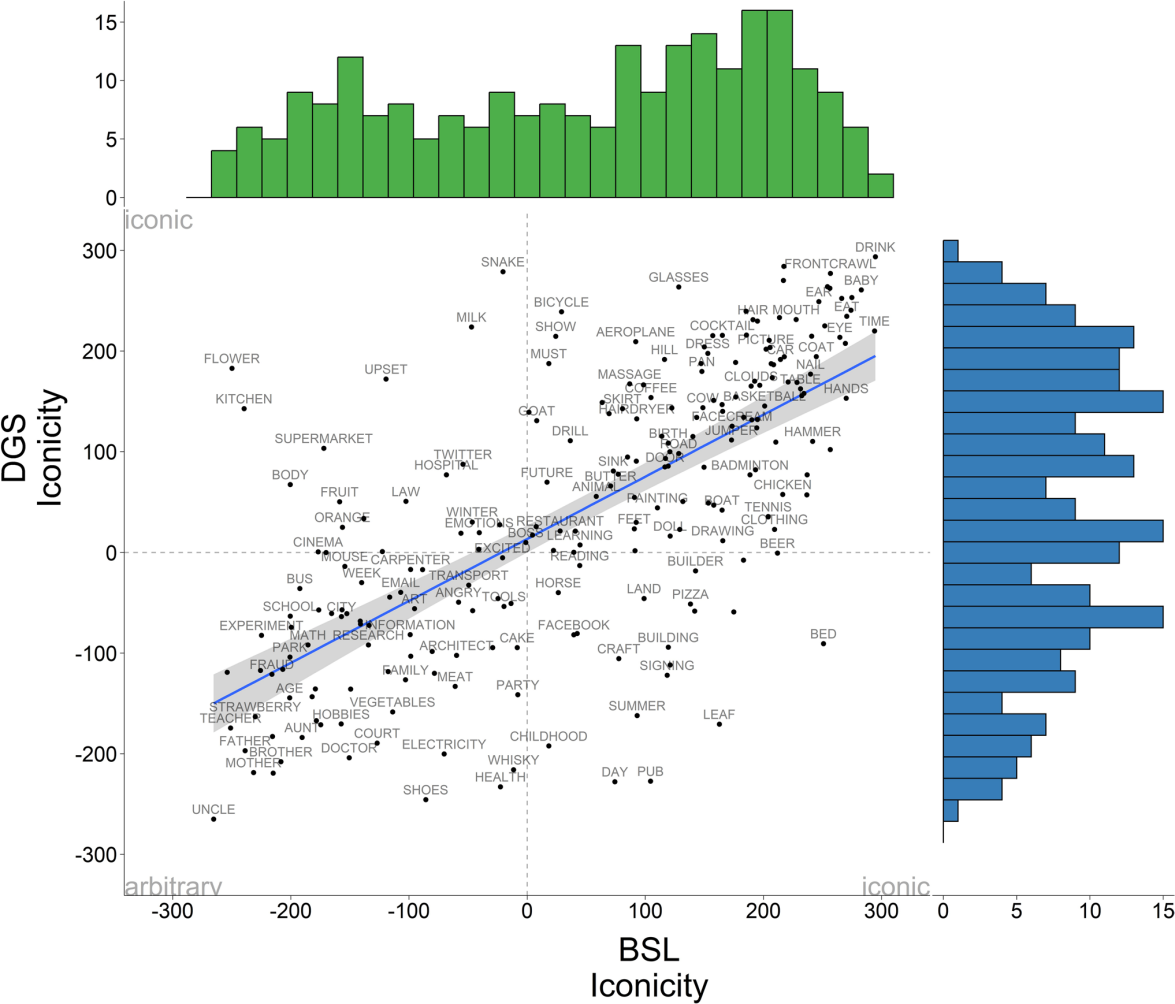
Correlation of mean iconicity ratings in BSL and DGS across all participants (deaf and hearing signers and non-signers) along the iconicity continuum (– 300 arbitrary, + 300 iconic). Frequency histograms showing the distribution of iconicity ratings are displayed on the horizontal (BSL) and vertical (DGS) axes

We estimated the mean iconicity ratings by computing the average rating for each concept across all participants regardless of whether they were deaf or hearing, signers or non-signers. Mean ratings were centred along a – 300 to + 300 scale so that positive values represent higher iconicity, negative values lean towards the arbitrary side, and zero denotes a mid-point between both ends. The mean iconicity ratings for all concepts were, for BSL: 47.56 (*SD* = 210.31) and for DGS: 43.14 (*SD* = 214.35). Figure 1 displays the correlation between iconicity ratings in BSL and DGS for all participants. Each label represents a concept and its location on the horizontal axis is the average BSL rating while its location with respect to the vertical axis is the average DGS rating. Frequency histograms of the distribution of iconicity ratings along the normalised scale are presented on the horizontal (BSL) and vertical (DGS) axis of the correlation plot. We found a strong positive correlation in iconicity ratings across both sign languages (*r* = 0.69, *t* = 14.61, *df* = 232,* p* < 0.001). The lower left side of the plot shows a concentration of signs with low iconicity ratings in both sign languages which have no evident link with the referent, such as kinship terms (UNCLE in DGS) or fingerspelled/initialised signs (e.g., FATHER in BSL). The upper right corner includes highly iconic concepts such as tools and actions depicting object manipulation (e.g., HAMMER, SAW, NAIL) or signs that touch or point to the referent (e.g., HAIR, EYE, TIME). Interestingly, we observe that these highly iconic signs have very similar forms in both sign languages, whereas signs low in iconicity are more likely to have different forms (Schiefner et al., under review). Regarding the distribution of the iconicity ratings for each language, we can see that BSL (horizontal axis) exhibits a slight bimodal distribution but with a more prominent bias towards the iconic end of the scale. DGS has a more even distribution of ratings along the scale but has more representation in the middle than at the lower end of the scale compared to BSL. The data show that both iconic and arbitrary signs are represented in the BSL and DGS lexicon, with a slight skew towards the iconic side.

**Fig. 2:**
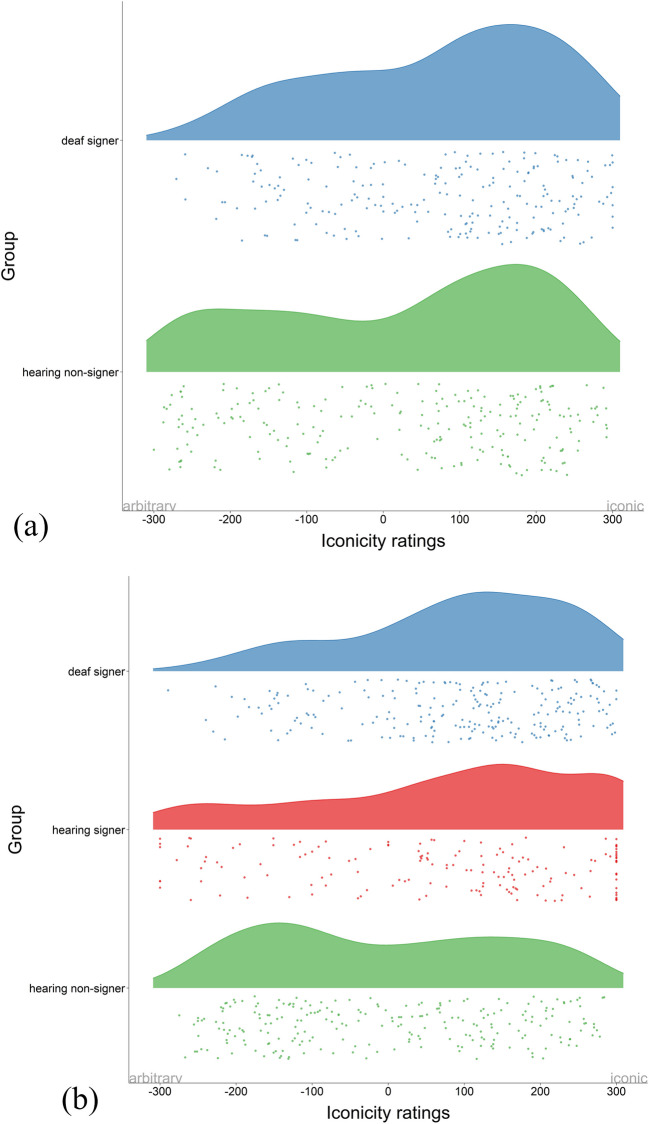
Raincloud plots displaying mean iconicity ratings in BSL (**a**) and DGS (**b**) across deaf signers, hearing signers, and hearing non-signers (note that there are no hearing signers in the BSL group). Wider areas of the raincloud plots represent a higher concentration of data points on the scale (– 300 arbitrary, + 300 iconic)

In order to understand how each group contributes to overall iconicity ratings and to alleviate concern regarding the unbalanced sample size across groups (deaf signers, hearing signers, and hearing non-signers), we created separate plots for deaf signers, hearing signers (only DGS), and hearing non-signers. Figure 2 displays ratings for each group across sign languages. The most prominent area of the rainclouds represents a higher concentration of data points along the continuum (negative values represent more arbitrary ratings and positive values more iconic ratings). For BSL (Fig. 2a), we can see that deaf signers’ ratings are skewed towards the iconic side of the scale and tail off gradually towards arbitrary values. Similarly, hearing non-signers’ ratings are also skewed towards the iconic end of the scale, but they display a more homogeneous distribution of data points from the central part of the scale towards arbitrary values at the low end of the scale. Regardless of whether they know a sign language or not, both groups perceive a large proportion of signs as iconic. For DGS, the profile of deaf signers’ ratings is very similar to BSL with a higher density of data points on the right side of the scale (more iconic) and the curve tailing off gradually towards arbitrary values. Hearing signers show a very similar pattern, though with a less prominent bias towards iconic values and a more even distribution of data points along the whole scale. German hearing non-signers exhibit a very different pattern from deaf and hearing signers in Germany (and the UK) because the largest concentration of data points is clustered towards the arbitrary end of the scale with a somewhat homogenous distribution along the iconic end. To sum up, with the exception of hearing non-signers in Germany, ratings of all groups are biased towards the iconic side of the scale.

**Fig. 3:**
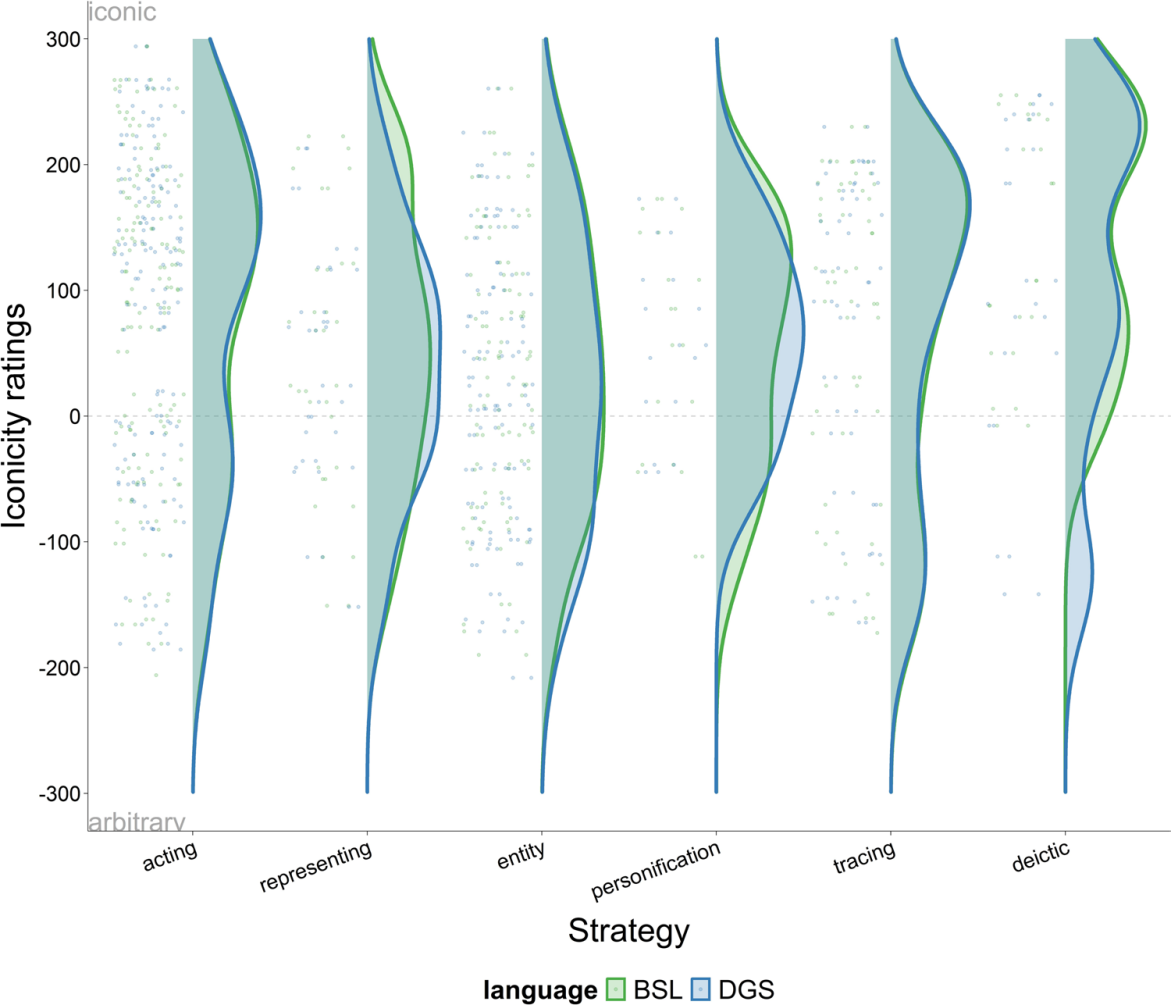
Violin plots displaying iconicity ratings per individual iconic strategy across all participants for BSL (*green*) and DGS (*blue*). Wider areas of the raincloud plots represent a higher concentration of data points on the scale (– 300 arbitrary, + 300 iconic)

We now turn to the relationship between iconicity ratings and the different iconic strategies. Given that iconicity ratings across participants are highly correlated in BSL and DGS and that the distribution of ratings is very similar across participants (except hearing non-signers in Germany) we present the results of all participants in the same graph. Figure 3 displays violin plots of the different strategies (i.e., acting, representing, entity, personification, tracing, and deictic) on the horizontal axis and iconicity ratings for the signs comprising each strategy on the vertical axis (– 300 arbitrary, + 300 iconic). As in the previous plot, each dot represents the mean iconicity rating of each sign across all participants and the width of the violin plot reflects the concentration of data points. BSL (green) and DGS (blue) are displayed on top of each other. Visual inspection of the data reveals that some strategies have the highest concentration of data points around the iconic (upper) end of the scale while other strategies are more evenly distributed along the whole continuum. More specifically, acting and entity, the most commonly used strategies in our dataset, show a more homogenous distribution along the whole scale, although signs using acting are slightly more skewed to the iconic side. In contrast, representing, personification, tracing, and deictic include most of the data points in the iconic region. Together, these data show that some strategies (acting and entity) are likely to be perceived as iconic as well as arbitrary, while other strategies (i.e., representing, personification, tracing, and deictic) are largely perceived as iconic.

**Fig. 4:**
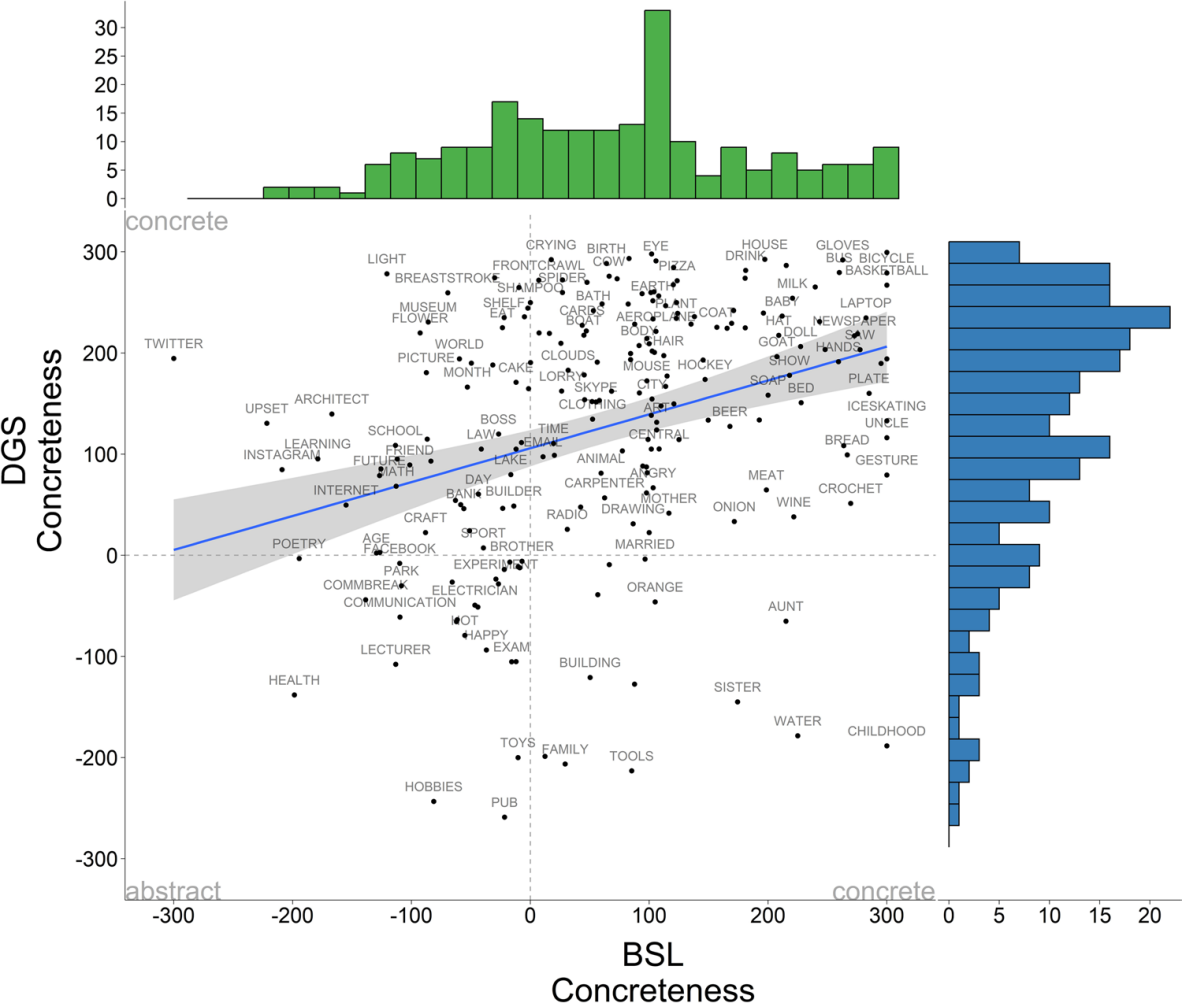
Correlation of mean concreteness ratings in BSL and DGS for deaf signers along the concreteness continuum (– 300 abstract, + 300 concrete). Frequency histograms showing the distribution of concreteness ratings are displayed on the horizontal (BSL) and vertical (DGS) axes. There is a higher concentration of data points in the concrete quadrant of the graph in both sign languages

**Fig. 5:**
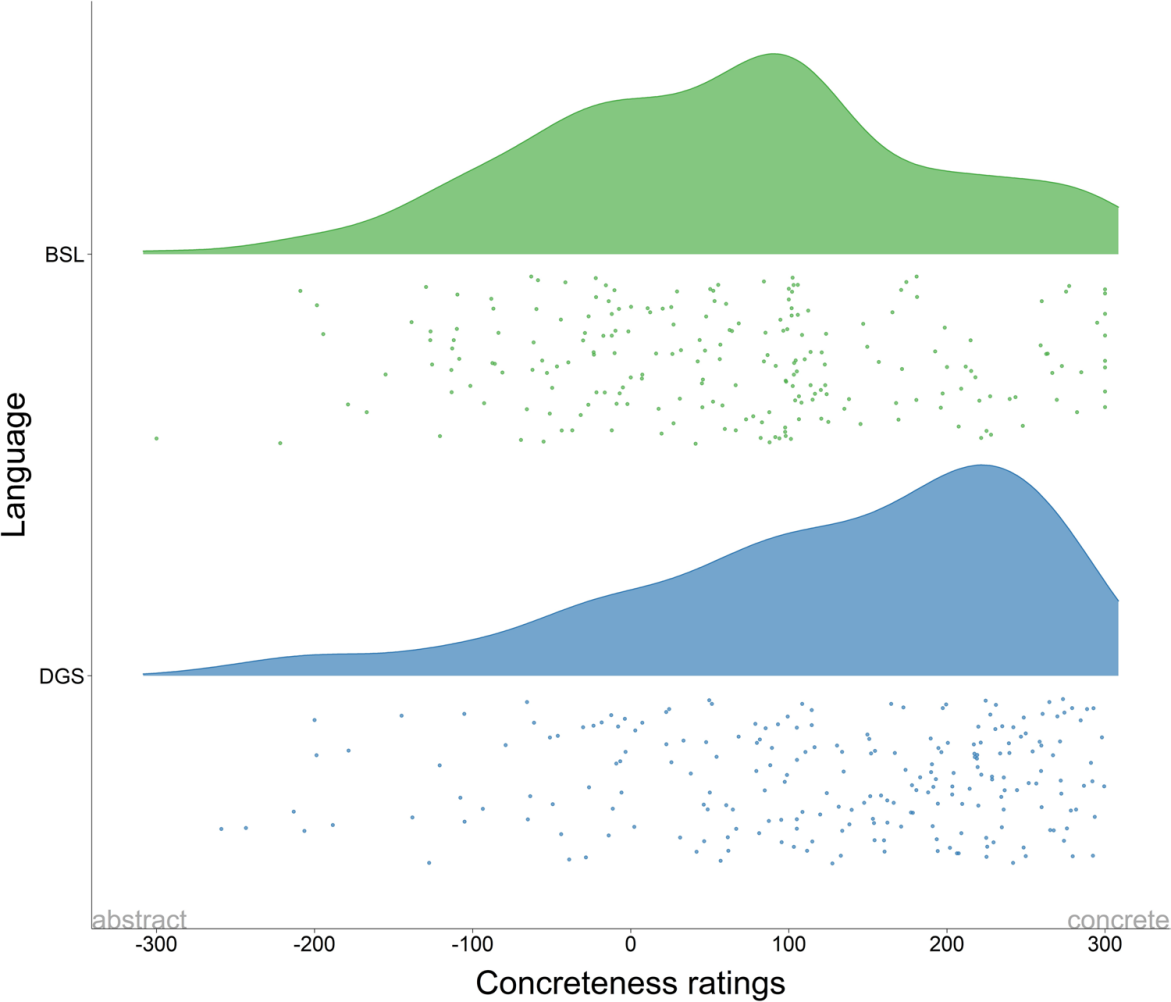
Raincloud plots displaying mean concreteness ratings in BSL and DGS for deaf signers. Wider areas of the raincloud plots represent a higher concentration of data points on the scale (– 300 abstract, + 300 concrete). In both sign languages, signs are perceived as more concrete, but the effect is more pronounced in DGS

Next, we estimated the mean concreteness ratings for each concept for deaf signers in both sign languages. Values are normalised on a scale ranging from – 300 (abstract) to + 300 (concrete). The mean concreteness ratings for all concepts were, for BSL: 109.78 (*SD* = 199.71) and for DGS: 118.79 (*SD* = 188.70). Figure 4 shows the correlation plot of concreteness ratings for BSL and DGS. Similar to the correlation of iconicity in Fig. 1, each label represents a concept and its relative location in the plot is the mean average of the rating in BSL (horizontal axis) and DGS (vertical axis). There is a moderate positive correlation in concreteness ratings in BSL and DGS with a high concentration of data points in the concrete quadrant of the plot (*r* = 0.32, *t* = 5.09, *df* = 219,* p* < 0.001). DGS is strongly skewed towards the concrete end of the continuum, whereas BSL is more evenly distributed along the scale with some prominence on the concrete end. For ease of visualisation, we created raincloud plots of concreteness ratings for BSL and DGS (Fig. 5), which show that indeed BSL has higher density around the mid-point of the scale with a slight skew towards concrete values while DGS has a strong skew towards the concrete end of the scale.

**Fig. 6:**
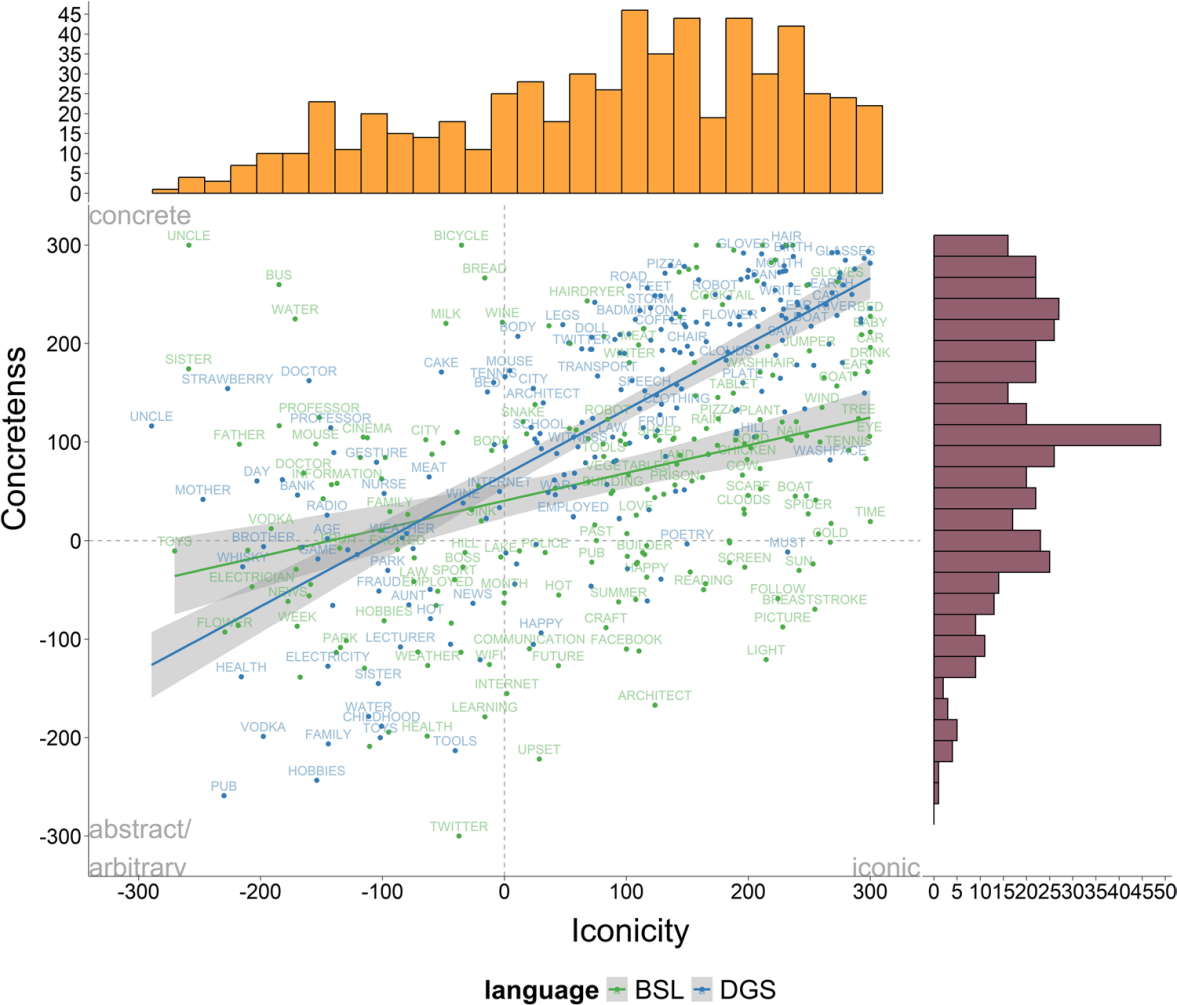
Correlation of signers’ mean iconicity ratings in BSL and DGS (– 300: arbitrary, + 300: concrete) by signers’ mean concreteness ratings in BSL and DGS (– 300 abstract, + 300 concrete). Colours mark ratings for BSL (*green*) and DGS (*blue*) signs, respectively. Frequency histograms showing the distribution of iconicity ratings are displayed on the horizontal axis and concreteness ratings on the vertical axis. In both sign languages, iconic signs tend to be perceived as more concrete, but this relationship is more pronounced in DGS than BSL

Finally, we conducted a correlation analysis to understand the relationship between concreteness and iconicity in both languages. Considering the differences between signers’ and non-signers’ perceptions of iconicity, we compute the correlation with ratings provided by signers only. In BSL, we found a moderate positive correlation between iconicity and concreteness ratings (*r* = 0. 34, *t* = 5.37, *df* = 216, *p* < 0.001). In DGS, we found a strong positive correlation between both variables (*r* = 0.73, *t* = 15.90, *df* = 223, *p* < 0.001). These results suggest that there is an overall trend for concepts with high concreteness ratings to receive high iconicity ratings, but this trend is more pronounced in DGS than BSL (see Fig. 6).

## Discussion

Our current understanding of the mechanisms behind language perception and production has relied on a multitude of efforts to document the psycholinguistic profile of thousands of words. These efforts, however, have focused primarily on written and spoken language, leaving the sign languages of deaf communities comparatively undocumented. In recent years, researchers have developed norms for multiple variables for many sign languages. This practice has fostered psycholinguistic experimentation, which has uncovered novel factors and effects in linguistic processes. Sign language research has thus been of paramount importance to better understanding language in all its facets and improving current theories of language use. To that end, the current study makes a small but significant contribution with a normed database for British Sign Language (BSL) and German Sign Language (DGS). We elicited signs from deaf signers and collected iconicity and concreteness ratings from deaf and hearing signers as well as hearing non-signers. In addition, we go beyond previous studies and also include different types of iconic strategies to explore their relationship with quantitative measures of iconicity. The direct cross-linguistic comparison of these ratings for the same concepts allows us to find general trends in the visual-manual languages of deaf communities as well as identify language-specific patterns.

**Fig. 7:**
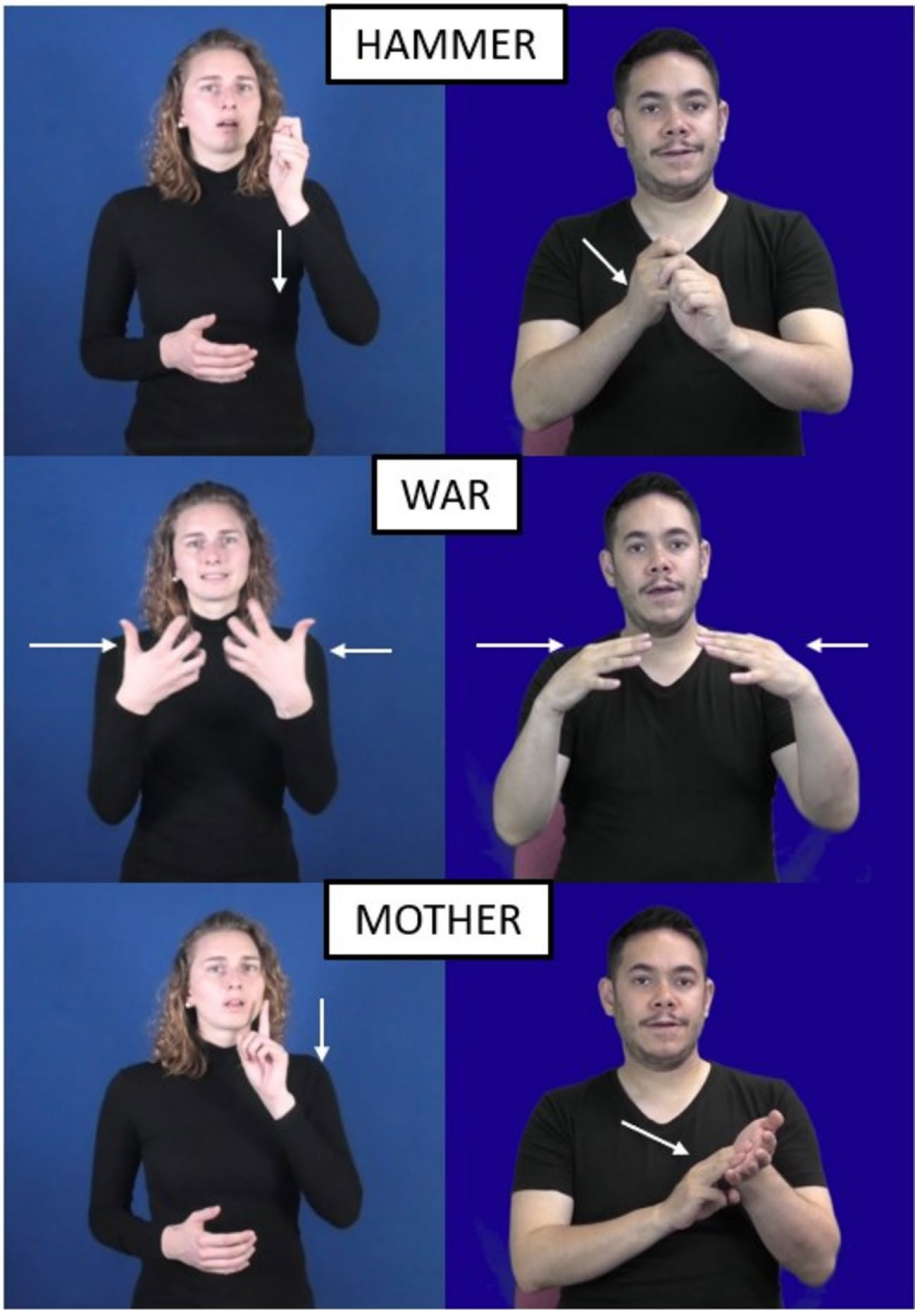
Examples in DGS (*left*) and BSL (*right*) showing systematic form-meaning mappings. The sign HAMMER (*top row*) represents the action of using a hammer with the hands. The sign WAR (*middle row*) uses the opposition of the fingers in both hands to represent the concept of war through a visual metaphor of opposing forces. For some concepts, such as MOTHER, there is no clear instantiation (DGS) or the sign relies on handshapes representing letters (e.g., M for MOTHER in BSL)

We found that there is a strong correlation between the iconicity ratings across all participants (deaf and hearing signers and hearing non-signers) in BSL and DGS. This high correlation could be explained by both sign languages making similar form-meaning mappings that are perceived in very similar ways across participants with different linguistic knowledge. Evidence to support this claim comes from our data showing that some concepts cluster systematically in clear regions along the iconicity continuum. As described in the Results section, we can see that in both sign languages, signs using bodily actions to represent tools (e.g., HAMMER), object manipulation (e.g., NAIL), and actions (e.g., SWIMMING), were located on the more iconic end of the scale. This was also the case for signs that touch or point to the referent (e.g., HAIR). When we look at the arbitrary end of the scale, we see a cluster of kinship terms (e.g., AUNT) or signs whose iconic motivation cannot be easily perceived (e.g., AGE). At the mid-point of the scale, we see signs that depict emotions which are typically articulated in the torso area (e.g., ANGRY, FRUSTRATED) or signs that express abstract concepts through metaphoric or metonymic relationships (e.g., WAR). See Fig. 7 for examples.

There is no doubt that iconicity is a subjective construal that varies depending on an individual’s lived experiences (Occhino et al., 2017). However, our study shows some commonalities in the way unrelated sign languages create form-meaning associations which may result in similar iconicity ratings by different populations. For instance, signs exploiting body schemas to represent actions or objects and signs that physically point to the referent create a more direct connection with the referent, so, not surprisingly, these yield the highest mean ratings in both BSL and DGS. We also observe some systematic mappings in the way signs exploit metaphor to represent less imageable concepts and which could result in very similar ratings. Interestingly, some of these metaphoric signs also have very similar forms across languages (e.g., WAR, FUTURE). Signs distributed in the arbitrary quadrant often exploit semiotic resources that create a seemingly distant connection with the concept it represents which result in lower ratings. This is the case with fingerspelled letters, which are manual representations of graphemes, but bear little resemblance with the referent (e.g., M for MOTHER in BSL). It is true that iconicity ratings reflect a subjective construct that depends on individual experience (Occhino et al., 2017), but we argue that there are systematic form-meaning mappings that result in a surprisingly similar distribution of ratings across these two unrelated sign languages. Granted, British and German participants are Western European cultures some shared history, but nonetheless, the affordances of the manual–visual modality constrain the way in which signs can represent concepts visually, and this results in systematic mappings between a manual component and its meaning. These systematic mappings may be perceived in similar ways (in varying degrees) by different groups regardless of their linguistic knowledge and thus result in similar iconicity ratings.

That being said, there are also numerous signs that differ significantly in their form-meaning mappings as well as their respective iconicity ratings. In the sign SUPERMARKET in DGS, for instance, two closed fists moving forward represent the pushing of a trolley, while the BSL sign represents the pulling items off the shelf and placing them into a trolley. In this example, the DGS sign received high iconicity ratings (133.33), while the BSL sign received a low rating (– 172.22). Similarly, the sign PUB in BSL represents someone drinking from a pint glass (high iconicity, 104.33) while the DGS equivalent consists of two V-handshapes one tapping on the other (low iconicity, – 232.43). These examples demonstrate that while there may be general shared commonalities in the way signs represent some concepts, in other instances, the associations are very distinct because they depict different semantic features of the referent or because they exploit different iconic strategies. The consequence is that the same concept receives very disparate iconicity ratings.

An important difference we observe in our data is that deaf and hearing signers across cultures exhibit different patterns in the distribution of iconicity ratings. For BSL, deaf signers and hearing non-signers show a similar pattern whereby iconicity ratings in both groups are highly correlated and largely skewed towards the higher (more iconic) end of the scale. For DGS, however, both groups behave quite differently; while deaf (and hearing) signers’ ratings are biased towards the iconic end of the scale, the ratings of hearing non-signers cluster towards the lower (arbitrary) end. This set of results supports past research on several dimensions. First, there are a handful of studies reporting that ratings from deaf signers and hearing non-signers go hand-in-hand and follow a similar distribution. For BSL, for instance, Ortega (2013) reports that ratings from hearing non-signers are highly correlated and follow the same distribution as those reported for deaf signers (Vinson et al., 2008). Similar findings have been reported for Israeli Sign Language (ISL) where ratings from deaf signers and hearing non-signers are also highly correlated and are skewed towards the iconic end of the scale (Fuks, 2023). In another study looking at American Sign Language (ASL), ratings from deaf signers and hearing non-signers also follow the same distribution, but an important distinction to BSL and ISL is that ratings from both groups for ASL are biased towards arbitrary values. Together, the general pattern we observe in this body of work is that within the same cultural group, ratings from deaf signers and hearing non-signers are (i) highly correlated and (ii) follow a similar distribution, even if their ratings differ along the iconicity continuum (e.g., more iconic for BSL and ISL; less iconic for ASL). This is not what we observe for DGS. Our results replicate findings by Trettenbrein et al. (2021) for DGS, who have also reported highly correlated iconicity ratings for both groups but with deaf signers’ ratings being also more skewed towards the iconic end of the scale and non-signers’ more skewed towards the arbitrary side.

**Fig. 8:**
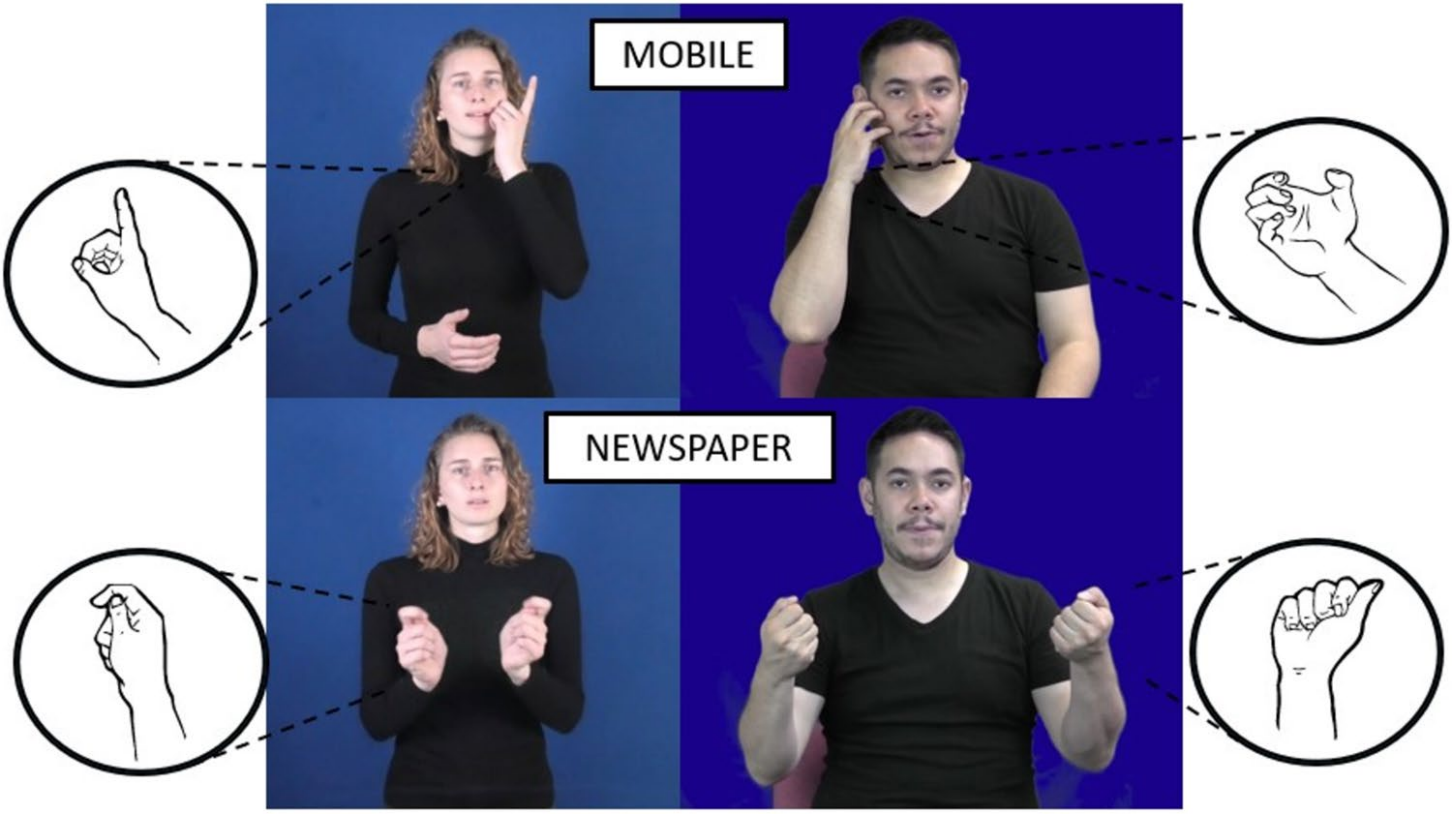
Signs MOBILE and NEWSPAPER in DGS (*left*) and BSL (*right*). The signs in both sign languages have the same iconic instantiation, but their handshapes are very distinct and may affect the perception of iconicity by deaf signers and hearing non-signers

A possible explanation behind the discrepancy between German deaf signers and hearing non-signers is that the specific phonological features of some DGS signs can eclipse their iconic instantiation, thus making it more inaccessible to non-signers. The sign NEWSPAPER in both BSL and DGS, for instance, derives from the action of opening a newspaper (Fig. 8). However, a difference between these signs is that the BSL sign is executed with a closed handshape that recreates the holding and opening of a newspaper. In contrast, DGS is executed with a double movement and a handshape that does not depict exactly how a person may hold a newspaper. The sign MOBILE recreates the holding of a device next to the ear, but while the handshape of the BSL sign depicts the holding of an object, the DGS sign has an extended index finger, possibly recreating the antenna of an old mobile phone. What we can see in these examples is that the constituents of the BSL signs may be more aligned with the iconic motivation of grasping or holding, and as a result, deaf and hearing signers possibly exhibit a similar distribution in their ratings. In contrast, the handshapes of some DGS signs represent a less canonical way of holding objects, so hearing non-signers may perceive these signs as less iconic.

The direct comparison of the form of signs for the same concepts allows us to see that even when signs have the same iconic instantiation (i.e., the holding of a newspaper or a mobile), there are marked language-specific phonological distinctions that could make them be perceived as less iconic by different groups. We should remember that iconicity is in the eye of the beholder and that different groups are likely to base their ratings on different schemas, linguistic knowledge, and lived experiences (Occhino et al., 2017; Sevcikova Sehyr & Emmorey, 2019). Only deaf signers have a manual phonological system to draw on, so they are capable of seeing the iconic motivation of a sign even if constituents (e.g., handshape) do not overlap with the actual referent. Hearing non-signers, in contrast, lack a manual phonological repertoire so they may be less able to perceive the iconic mapping when signs do not have a strong resemblance with the referent. In our study, German hearing non-signers may not have perceived to the same extent the iconic motivation of signs when these presented unexpected forms (e.g., an extended index finger to depict the holding of a mobile). Perhaps there is more form overlap between BSL signs and their referents (or with non-signers’ gestures) and as a result the ratings of deaf signers and hearing non-signers follow a more similar distribution.

These results replicate past research showing the strong correlation between iconicity ratings of deaf signers and hearing non-signers (Fuks, 2023; Ortega, 2013; Sevcikova Sehyr & Emmorey, 2019; Vinson et al., 2008), but they also suggest that language-specific features of the target sign language may be an important factor that dissociates ratings across groups. It may be possible to predict that ratings from deaf signers and hearing non-signers may be more dissociated if the constituents of signs are more phonologically complex or distinct from non-signers’ motor or gestural schemas. Our findings further support previous claims that while iconicity ratings may have similar distributions across different groups, they are heavily mediated by individuals’ experience with a language (Occhino et al., 2017; Sevcikova Sehyr & Emmorey, 2019).

Our study shows that the distribution of iconic strategies and iconicity ratings in both sign languages is very similar. The most common strategy in both sign languages is *acting* and its iconicity ratings are evenly distributed along the whole continuum, albeit slightly skewed towards the more iconic side of the scale. Signs using this strategy can represent signs with highly iconic ratings through the direct representation of actions (e.g., DRINK), objects associated with actions (e.g., HAMMER); or signs with lower ratings through metaphoric/metonymic depictions (e.g., WAR). These results add to the growing body of work showing that iconic forms using the *acting* strategy are perceived as more iconic than other strategies (Fuks, 2023; Ortega & Özyürek, 2019b; Sevcikova Sehyr & Emmorey, 2019). However, we also find that signs using the *acting* strategy can receive low iconicity ratings if the meaning associated with the referent is abstracted from the bodily action it depicts. That is, the body can be used to represent referents that are grounded to the bodily action represented in a sign (e.g., holding an imaginary cup for DRINK) but it can also be used to represent concepts that are more detached from sensory experience (e.g., the action of writing to represent STUDENT in DGS). The versatility of the *acting* strategy to use the body to represent a wide range of concepts makes it a productive mechanism in the BSL and DGS lexicons, and may be the reason why it is the most common strategy in both languages. The body is our primary vehicle to interact with the world and thus the *acting* strategy could be regarded as a ‘default’ to create manual iconic representations, making them more easily recognisable (van Nispen et al., 2014, 2016).

A very similar pattern was observed in *entity*, the second most common strategy in our study. Signs using this strategy are much more evenly distributed along the iconicity scale, more so than *acting*, because we can see a denser concentration of signs on both ends of the scale. The *entity* strategy uses different hand configurations to mimic a visual representation of the referent or events associated with it. For instance, the signs CITY and BANK in DGS make use of an open-claw handshape facing down to represent a generic building which allows for multiple conceptualisations. Similarly, in DGS, the signs READING and MUSEUM use a V-handshape to represent a person’s eye gaze. In READING, the sign represents the actual action of reading, whereas MUSEUM depicts an event typically associated with museums. The *entity* strategy is unique in that it does not rely so much on motor schemas to create analogies with a referent, but rather it exploits visual features that depict or are associated with a referent. As such, these specific features allow for a wide gamut of iconic and metonymic associations. The rest of the strategies, *representing*, *personification*, and *deictic,* can be set apart from *acting* and *entity*. Their iconicity ratings tend to be clustered on the iconic end of the scale, and they are not as commonly observed in our sample. The nature of these strategies grounds them more clearly to specific referents and thus there is a more direct link with the concept they represent. A prediction that could be addressed in future cross-linguistic studies in different sign languages is that *acting* and *entity* strategies are likely to receive a broad range of ratings along the iconicity scale, whereas the rest will be more skewed towards more highly iconicity values.

We now turn to our analyses on concreteness. Similar to the results for the degree of iconicity, we find that ratings for concreteness are highly correlated in both sign languages. However, an important observation is that compared to spoken languages, our ratings seem more skewed towards the concrete end of the scale. Researchers have reported large normed databases of spoken/written languages like Dutch (Brysbaert et al., 2014a, b) and English (Brysbaert et al., 2014a, b), where most words cluster towards the abstract end of the scale. Concreteness ratings for Italian have shown a slightly different profile in exhibiting a bimodal distribution with prominent peaks on the concrete and abstract ends of the continuum (Montefinese et al., 2023). In our study, in contrast, most signs are skewed towards the concrete end of the scale: BSL has an almost normal distribution with a significant trend towards concreteness, and DGS is strongly skewed towards the concrete side of the scale. In other words, there is a strong correlation between the ratings in these two languages which (i) are overall biased towards concreteness, but with (ii) DGS being perceived as more concrete than BSL.

One possible explanation behind the differences in concreteness ratings for spoken languages is that the manual-visual channel of expression of sign languages allows for the embodiment of concrete and abstract concepts making them more grounded to our sensorimotor experiences. Borghi et al. (2014) have challenged current theories explaining abstract representation by exploring Italian Sign Language (LIS) as a testament of the embodiment of typically assumed abstract concepts. Some theories argue that abstract concepts evoke introspective states (Barsalou, 1999), but in LIS, as well as other sign languages, these states can be depicted through metaphors linked to bodily actions. For instance, mental verbs in LIS treat thought as a concrete entity and as such signs like UNDERSTAND and FORGET represent the catching or releasing of said entity from the head. Similarly, in our dataset as well as in other sign languages (Meir, 2010), the sign LEARN represents bringing something towards the head (BSL) or ideas as objects (DGS). In line with the examples in LIS, the mental verbs in our study could have been perceived as more concrete given the metaphoric mapping between an action and the intended meaning. A related explanation is that sign languages depict emotions in more transparent ways and this in turn could result in more concrete ratings compared to spoken languages. Our study includes several emotion signs (e.g., ANGRY, FRUSTRATED, EMOTIONS), which are typically articulated at the chest/torso and are accompanied with matching non-manual features (i.e., an angry face for ANGRY). Accordingly, these concepts receive high concrete ratings, which goes against what is typically observed in spoken languages (e.g., the concreteness ratings of the sign ANGRY in BSL – 103.3) and DGS (– 66.71) show that these concepts are regarded as more concrete while their English (133.93) and German (75.86) counterparts are considered to be more abstract.

Further, we also noticed that the correlation in concreteness between spoken languages (*r* = 0.92, *p* < 0.001) is stronger than between sign languages (*r* = 0.32, *p* < 0.001), which could be attributed to differences in the spoken vs. written vs. manual modalities of language. Concepts that are typically regarded as highly abstract in spoken languages acquire a more concrete dimension in sign languages through the metaphoric representation of mental events and emotions. These dimensions are not absent in speech, as they can be expressed in their co-occurring gestures, but these embodied notions of abstract concepts cannot be captured in the arbitrary nature of written words, at least not to the same extent as in sign languages. This being said, it is important to exercise caution because our sample size is significantly smaller than normed databases of spoken languages (i.e., 234 signs in our study compared to 30,000–40,000 words in studies in spoken languages, e.g., Brysbaert et al., 2014a, b). Nonetheless, our data add to the growing body of evidence that the manual-visual modality highlights important differences that affect psycholinguistic variables such as concreteness.

Another important contribution of our analyses is that they shed light on the relationship between iconicity and concreteness ratings. Previous studies have shown mixed results regarding the relationship between these variables. For instance, some studies have found no reliable association between iconicity and concreteness ratings for English words (Winter et al., 2017). In contrast, a strong negative correlation has been reported for Spanish words (Hinojosa et al., 2021), whereas a strong positive correlation was found between iconicity and concreteness in phrasal compounds in Mandarin consisting of a prosaic and an ideophonic component (Van Hoey et al., 2024). Our own data show a moderate and strong positive correlation for BSL and DGS, respectively, between iconicity and concreteness. Together with the findings from Van Hoey et al. (2024), our findings support a pronounced association between iconicity and concreteness, particularly in structures that allow for vivid sensory representation. Overall, these findings support the notion that iconicity and concreteness are different constructs, and that the nature of their relationship may be modulated by the construal of the lexicon. For lexicons that support the depiction of sensory imagery, as in the case of languages in the visual modality or languages with ideophonic word classes, we may expect iconicity and concreteness to be positively correlated. In addition, the findings point to the importance of investigating the relationship between different variables in psycholinguistic research across word types, languages, and modalities.

## Conclusion

In this cross-linguistic study, we find that unrelated sign languages show strong similarities in their iconicity ratings that can be attributed to the shared manual-visual modality (and perhaps to some extent close cultural relatedness). At the same time, we consider that language-specific features of each language (e.g., the handshape of iconic signs) may obscure the iconic instantiation of some signs and this could result in a different distribution of ratings across different populations. The perception of iconicity is a subjective process and thus it will be heavily mediated by linguistic knowledge of a sign language. An important contribution of our study is that we show that looking at the relationship between iconicity ratings and iconic strategies reveals important insights into the distribution of signs along the continuum. We argue that concreteness ratings are skewed towards the concrete end of the scale because of the embodied, iconic, and metaphoric nature of the manual-visual channel. These results strengthen our call to take serious account of the different types of iconic strategy in the psycholinguistic profile of signs because, as the data show they have a distinct distribution in ratings and thus they may evoke different responses in linguistic processes. Only by including sign languages in our research agenda can we develop a comprehensive model of language processing.

## Data Availability

Data, coding schemes, ratings, code, statistical analyses, signed instructions (and their translations), and videos of the signed concepts in BSL and DGS can be found in our open access repository: 10.17605/OSF.IO/JKW8P.

## Appendix 1

English translation of the instructions for the iconicity ratings.

In this experiment, you will see short videos of single signs in British/German Sign Language with their English translation. Some signs look like what they refer to. These are called iconic. Other signs do not show any similarity, and they are called iconic signs. Your task in this experiment is to rate how much each sign looks like what it means on a scale, using a slider. For example, the signs for PUSH is highly iconic. The sign for PUSH looks like pushing someone. The sign for NAME, on the other hand, does not look like what it means at all. It is arbitrary. Please rate each sign’s iconicity using the slider. For example, PUSH is very iconic, while NAME is arbitrary. Of course, there are also signs that fall somewhere in between. Those should be rated between the two extremes. Please use the whole range for this. Don’t worry about selecting the same number multiple times, as long as you are giving your honest opinion. If you feel that you are unable to provide any estimate, please select “no response”. Don’t think about your answers for too long. There are no right or wrong answers. We are interested in your personal opinion.

## Appendix 2

English translation of the instructions for the concreteness ratings adapted from (Brysbaert et al., 2014a, b).

In this study, you will be presented with individual signs. Your task is to rate how concrete the concept is to you using a slider to go from concrete to abstract. For example, the concept SOFA is very concrete. You can see it, touch it, and experience what sitting in it feels. You can experience a SOFA with all your senses. SOFA would therefore be rated as very concrete. The concept HOPE, meanwhile, is very abstract. How we describe hope depends on the context. While we can associate sensory experiences with the concept, we are unable to experience the concept itself. HOPE would therefore be rated as very abstract. Of course, there are also signs that fall somewhere in between. Those should be rated between the two extremes. Please use the whole range from concrete to abstract for this. If you feel that you are unable to provide any estimate, please select “no response”. Don’t think about your answers for too long. There are no right or wrong answers. We are interested in your personal opinion.

## Footnotes

1 The present study was carried out at the start of the COVID-19 pandemic (March 2020 to June 2023), which impacted many of the original features of our study, including the number of participants. While we could only recruit nine deaf signers, they produced a sufficient number of tokens for analysis.

2 The link to our OSF repository is: 10.17605/OSF.IO/JKW8P.

3 Note that some authors use the label *handling* to refer to *acting* but nonetheless these terms refer to the strategy where the body represents the body.

4 A large number of participants joined the surveys but were excluded due to implausible responses (e.g., random guessing on quality checks, extremely limited ranges with less than 100 points between the extremes and implausible response times). We are therefore only reporting data on the raters that passed our quality checks.

5 Based on simulations of different numbers of ratings per item for published datasets of iconicity ratings, Motamedi and colleagues suggest that ten ratings per item is reliable (Motamedi et al., 2020). In our dataset, the iconicity ratings are based on numbers well within the suggested threshold. This is less clear, however, for some of our concreteness ratings, in particular for BSL. While there is no empirical evidence for the necessary number of ratings per item for reliable concreteness ratings in sign languages, this caveat should be kept in mind.
